# The resiliency of noncommunicable diseases services during the public health crisis: a lesson from Bangkok, Thailand

**DOI:** 10.1186/s12913-023-09400-z

**Published:** 2023-04-26

**Authors:** Siriwan Pitayarangsarit, Nanapas Bhagaman, Korravarn Yodmai, Nattaya Thangsirikul, Mathuros Tipayamongkholgul

**Affiliations:** 1 Division of Non-communicable Diseases, Department of Disease Control, Ministry of Public Health, Nonthaburi, Thailand; 2 International Health Policy Program, Ministry of Public Health, Nonthaburi, Thailand; 3 Department of Epidemiology, Faculty of Public Health, Mahidol University, 420/1 Ratchawithi Road, Ratchathewi, Bangkok, 10400 Thailand; 4 Department of Family Health, Faculty of Public Health, Korravarn Yodmai, Mahidol University, 420/1 Ratchawithi Road, Ratchathewi, Bangkok, 10400 Thailand

**Keywords:** Non-communicable Diseases Services, Resiliency, Bangkok, COVID-19, Diabetes Mellitus

## Abstract

**Background:**

The healthcare services for non-communicable diseases (NCD) are commonly affected by public health crises like the COVID-19 pandemic. During the pandemic, all healthcare facilities in Bangkok had been overwhelmed by the extreme caseload of COVID-19. Health service resiliency is crucial for the continued service of healthcare facilities post pandemic. This study aims to explore the impacts of COVID-19 on NCD service disruption and addressed the resilience of healthcare services at the operational level.

**Methods:**

Healthcare facility-based surveys and in-depth interviews were conducted among representatives of the facilities in Bangkok from April 2021 to July 2021. The web-based, self-administered questionnaire, was sent to directors or authorities of all healthcare facilities in Bangkok Thailand (n = 169). Two healthcare facilities from three levels of health services were purposively selected. The directors or medical doctors and nurses who are in charge of the NCD service, and working at the six selected health facilities, were invited to participate in the in-depth interviews. Descriptive statistics were used to analyze the survey data, and thematic analysis was used to analyze the data from the in-depth interviews.

**Results:**

The impact of COVID-19 on NCD service disruption in the second wave (2021) was more severe than in the first wave (2020). The main reasons for NCD service disruptions are insufficient staff, and the closure of some services offered by the healthcare facilities. Surprisingly, both the budget and medical supply for healthcare facilities in Bangkok are less affected by the COVID-19 pandemic. Our study revealed resilience capability i.e. absorptive, adaptive, and transformative capabilityamong the healthcare facilities that provide a continuum of care by increasing availability and accessibility to healthcare services for chronic illness as DM. The service disruption in Bangkok may alter from other provinces because of variations in COVID-19 incidence and health services contexts.

**Conclusion:**

During the public health crisis, using affordable and common digital technologies to ensure DM patients can access a continuum of care and providing alternative services such as mobile medical laboratories, medication delivery, and medical refill at drug stores can increase consistent monitoring of glycemic levels and use of prescribed medication.

## Background

Noncommunicable diseases (NCDs) mainly contribute to premature mortality worldwide, about 71% of overall mortality [1]. To control NCDs, a continuum-of-care approach has been widely implemented to control NCD at each stage of the disease progression [2]. In particular, patients with NCDs such as diabetes mellitus (DM), require long-term care i.e. regular clinic visits, monitoring biomarkers such as blood glucose level and kidney function, constant availability and uptake of prescribed medication, and counseling for lifestyle modification to prevent sequelae of the disease [3].

COVID-19 has profoundly placed negative impacts on social, economic, and public services worldwide [4–9]. In Asia, many countries reported income declining by 50 − 70% [4, 5]. Many countries implemented public health measures to fight against COVID-19 that have devastated countries’ economies such as lockdowns and the closure of non-essential businesses [4, 8, 9]. These measures increased the economic disparity within and among countries, however, different economicabilities to comply with the measures between high- and low-income individuals are likely related to the risk of COVID-19 infection [4, 6]. Crowded and high socioeconomic areas are strongly associated with a higher incidence of COVID-19 [7, 8], coupled with severely impacted health systems [9, 10].

Additionally, those public health measures against COVID-19 such as voluntary social distancing, stay-at-home orders, active case-findings, and vaccination programs also indirectly affected health service systems, in particular NCDs services [9]. NCDs patients are most vulnerable to severe illness of COVID-19 and are more likely to have worse clinical outcomes compared to those with preexisting diseases [10–13]. These critical linkages between COVID-19 and NCDs may likely be related to hyperinflammatory responses. Also, medications-related hypertension and DM-induced severity of COVID-19 among NCD patients [11–14].

At the beginning of 2020, the first case of COVID-19 outside of China was reported in Bangkok, Thailand, and has led to several COVID-19 surges until 2022. From May 2021 to December 2021, the daily confirmed cases of COVID-19 ranged from 1,000 to 17,000 cases [15]. Bangkok, the capital city of Thailand, reported the highest COVID-19 caseloads and deaths in the country [15]. The escalating COVID-19 numbers in Bangkok have drawn all public health attention to flattening the COVID-19 curve. Adjusting public health and social measures such as workplace closures, physical distancing, and gathering size restrictions in the context of ongoing uncertainties highly affects the care of NCD patients and the complexity of designing healthcare services. These constraints and the vulnerability of patients with NCD to the severity of COVID-19 infection cause this complexity in designing the NCD services during the COVID-19 crisis. Previous studies in low-and middle-income countries (LMIC) reported that NCD patients’ accessibility to healthcare services are disrupted by the virus, including follow-up visits, biomarker testing, and receiving prescribed medication during the COVID-19 pandemic [3, 16, 17]. Moreover, public health measures for COVID-19 affect patient lifestyles and increase complications among NCDs patients [16]. This condition can lead to increased complications and mortality in patients with NCD in LMICs. The impact of COVID-19 on the provision of NCDs services i.e. cancer and cardiovascular disease were reported in the Southeast Asia region [10, 18] but have not yet explored the resilience of healthcare services [9].

During public health crises such as the COVID-19 pandemic, the resilience of the healthcare services is a crucial character, particularly in NCDs that require long-term services. Health system resilience is known as the ability of health systems to absorb, adapt, or transform to maintain essential functions when stressed or shocked [19–21]. The resilience of healthcare services focuses on three capacities i.e. absorptive is a capacity to retain core function under the public health crisis using existing available resources, adaptive is a capacity to deploy additional resources and/or reconfiguration of resources, and transformative is a capacity to radically change system structures to achieve goals of service [9, 18–21]. Among NCDs, hypertension and diabetes mellitus are a crucial problem in Thailand, however, in 2021, hypertension patients presented better health outcome compare to diabetes mellitus [22]. Only 30% of DM patients can control their blood sugar levels, and none of DM suspected cases were diagnosed [22]. The poorer performance of DM services than hypertension during COVID-19 pandemic may be related to DM required professional services such as examination for blood sugar level and hemoglobin A_1_C (HbA_1_C) [9, 11, 14].

Understanding the impact of COVID-19 and the resilience capabilities of the healthcare service during the public health crisis can provide insightful information for the public health authorities to formulate a strategic plan for healthcare services to operate during a future public health crisis. This study used both quantitative and qualitative methods to explore the impacts of COVID-19 on NCD services, address the resilience of healthcare services at the operational level, and define optimal adaptive NCD services in primary, secondary, and tertiary care services in a megacity like Bangkok.

## Methods

### Study settings, study population, and data collection

To explore the adaptation of NCD services in Bangkok during the COVID-19 pandemic from 2019 to 2021, we applied the mixed method and conducted it in two phases. Participants received oral and written information about this research, and informed consent was obtained from the participants prior to the study.

In phase I, we aimed to measure the impacts of COVID-19 on NCD services. We sent a self-administered web-based structured questionnaire to all private healthcare facilities and all healthcare facilities under the jurisdiction of BMA, Ministry of Public Health and Minister of Education (Medical Colleges) situated in Bangkok from April to July 2021.

In phase II, we intended to discover insight and understanding ofthe resilience capability of healthcare services during the COVID-19 pandemic by applying the Blanchet’s resilience framework [19] via a case-study of outpatient services for diabetes mellitus (DM). We therefore purposively selected six healthcare facilities from three levels of Thailand’s public health services i.e. two BMA health centers (primary care unit), two secondary care facilities (hospitals with less than 500 beds), and two tertiary care facilities (hospitals with more than 500 beds and excellent centers). The interviewees were recommended by the division of Noncommunicable diseases, department of Disease Control, Ministry of Public Health, and Bureau of Medical Services, Bangkok Metropolitan, Thailand. We then purposively selected key informants from 2 groups: (1) the director of the Primary Care Commissioning Cluster (PCC), National Health Security Office to understand changes in the national universal health coverage policy to support healthcare services during a COVID-19 pandemic; (2) Targeted healthcare practitioners including a head of the NCD clinics, a head of the out-patient division, a physician or a nurse who provides services at the NCD clinics at least 2 years and voluntarily participated the in-depth interview. Due to the public health measures to control COVID-19 during the period of our study, the interviews were conducted via online discussions. The virtual in-depth interviews were arranged, and permission was obtained from each of the key informants for audiotaping the interview before starting. MT, NB, and KY conducted the virtual in-depth interviews from July 2021 to August 2021. All interview records were transcribed non-verbatim by NB.

### Research tools

For phase I, the web-based structured questionnaire was used to explore the impacts of COVID-19 on NCDs services comprised of four sections: (1) Responsible position and location of the healthcare facilities 4 items; (2) Facility policies and patient guidelines for NCDs services during the COVID-19 pandemic comprised 4 items, (3) Impacts of the COVID-19 pandemic on NCDs services, screening, treatment and care, patients’ follow-up appointment comprised 10 items of 4 scales response, and (4) Services adaptation and results comprised 7 items of multiple choices. We classified the questionnaire into two waves of the COVID-19 pandemic, the first wave from December 2019 to December 2020 (R_0_ = 9), and the second wave from January to April 2021(R_0_ = 35) [1, 2].

For phase II, we developed the in-depth interview guideline complying with the resilience capability framework [19] to aggressively interrogate (1) how the selected healthcare facilities have continued the DM services, (2) how they reconfigured the resources or adjusted service during the pandemic, and (3) how they created an innovation or long-lasting changes in healthcare services. These questions further explored the adaptation-related internal factors such as staffing, financing, leadership and governance, health information system, and adaptation-related external factors such as national and BMA policy, and lastly availability and accessibility of DM services.

All data collection was conducted after the study protocol was approved by the Ethics Committee, the Institute for the Development of Human Research Protections (COA No. IHRP 2,021,002, 14 January 2021).

### Data analysis

The survey data was analyzed by frequency and percentage using Microsoft Excel 2019 (Mahidol University). Thematic analysis was undertaken and audio recordings were reviewed against the non-verbatim transcription of in-depth interviews by researchers. To ensure the validity, the theme development was evaluated using feedback from the stakeholder forums (including 1) the director of National Health Security Office for Bangkok Section, 2) the deputy director of the Noncommunicable diseases division, MoPH, 3) the deputy director of the Bureau of Medical Services, Bangkok Metropolitan Administration, 4) Directors and Heads of the NCD clinics of selected health services located in Bangkok and 5) researchers) on the model of non-communicable disease clinic services in Bangkok under emergency situations on September 16, 2021, by the International Health Policy Program, Thailand (IHPP) and Division of Noncommunicable Diseases, Department of Diseases and Control. An agreement on final theme categorization was discussed to increase the reliability.

## Results

### Phase I

We sent the web-based structured questionnaire to 169 public and private healthcare facilities. Of these, 81 healthcare facilities sent the questionnaire back to us (response rate = 47.9%). Of all the respondents, 71.6% were from the BMA health center, the respondents were ages 26 to 60 years old, 90% of the representative were female and half of them were in charge of the NCD clinic (Table 1).

**Table 1:** Characteristics of the healthcare facilities’ representative

Characteristics	Characteristics	Number (n = 81)	Percentage
Types of healthcare facility	BMA Health center	58	71.6
	Private hospitals	15	18.5
	General hospital	5	6.2
	University hospital	3	3.7
Age group (years)	26–40	30	37.0
	41–50	25	30.9
	51–60	26	32.1
Sex	Female	73	90.1
	Male	8	10
Job title	Head of the NCD Clinic	42	51.9
	Head of the out-patient division	14	17.3
	Director	7	8.6

Among 81 healthcare facilities, 80.2% and 77.8% provided NCD services as usual in 1st wave and 2nd wave, respectively. However, about 38% and 44% of them reported a moderate impact of COVID-19 on NCDs services, and 29.6% and 39.5% of them reported severe and extremely severe COVID-19 impact on NCDs service in 1st wave and 2nd wave, respectively (Table 2). About half of the respondents reported impacts from COVID-19 on the staff of NCDs clinics. During the 1st and the 2nd wave, NCDs clinic staff were deployed to provide COVID-19 relief (59.2% and 77.8%, respectively). In terms of budget, it represented a lower percentage in reduction and was inadequate during the 2nd wave in comparison to the 1st wave (3.7% against 2.5%). The most significant difference between waves was found in the allocation of medical supply, which increased by 3.7% from the 1st wave to 6.2% in the 2nd wave. (Table 2)

**Table 2:** Frequency of Impacts COVID-19 on NCD Clinic by the Epidemic Waves

Impact of COVID-19 on NCD Clinics		1st wave n = 81			2nd wave n = 81	
		n	%		n	%
**Service delivery**						
Continuity NCD services		65	80.2		63	77.8
Limit hours of NCD service		11	13.2		11	13.6
Limit days of NCD service		5	6.2		5	6.2
Closing of NCD services		1	1.2		2	2.5
**Level of COVID-19’s impact on NCD service**						
Not impact		6	7.4		4	4.9
Mild impact		20	24.7		9	11.1
Moderate impact		31	38.3		36	44.4
Severe impact		20	24.7		22	27.2
Extremely server impact		4	4.9		10	12.3
**NCD staff**						
NCD clinic staff continue usual tasks		55	67.9		47	58.0
NCD-related clinical staff partially deployed to partially provide COVID-19 relief		35	43.2		43	53.1
NCD-related clinical staff partially deployed to fully provide COVID-19 relief		4	4.9		5	6.2
All NCD-related clinical staff deployed to partially provide COVID-19 relief		9	11.1		13	16.0
All NCD-related clinical staff deployed to fully provide COVID-19 relief		0	0		2	2.5
**Allocation of medical supply**						
Same as usual		61	75.3		60	74.1
Reduction but adequate		13	16.0		12	14.8
Reduction and inadequate		3	3.7		5	6.2
Holding medical supply allocation		4	4.9		4	4.9
**Budget allocation for NCD service**						
Same as usual		64	79.0		61	75.3
Reduction but adequate		9	11.1		11	13.6
Reduction and inadequate		2	2.5		3	3.7
Holding budget allocation		6	7.4		6	7.4

Considering the essential care of NCDs such as diagnosis, treatment and care, follow-up clinical outcomes, behavioral change counseling, continuity of medication, and home visits, we found less than 25% of the healthcare facilities could provide the essential services by 95% of the target population. In particular, less than 20% of healthcare facilities achieved 95% target population of follow-up clinical outcomes, and behavioral change counseling in both epidemic waves. Continuity of medication showed that only 24.7% of healthcare facilities were able to provide their service above 95% as planned in the 1st wave and it was likely to decrease by 21.3% in the 2nd wave. We found that only 10% of healthcare facilities completed 95% of community-based services plans such as NCDs screening and home visits of plans (Table 3) Obviously, the 2nd wave expressed a more severe unmet target of the essential services than the 1st wave (Table 3).

**Table 3:** Impacts of COVID-19 on NCD services by Services Domain

Service domains	% of the target group received service as a plan		1st wave n = 81			2nd wave n = 81	
			n	%		n	%
**Diagnosis**	> 95%		18	23.4		17	22.7
	75–95%		22	28.6		20	26.7
	50–74%		18	23.4		18	24.0
	< 50%		13	16.9		14	18.7
	Not available		6	7.8		6	8.0
**Treatment and care**	> 95%		20	25.6		18	24.0
	75–95%		21	26.9		22	29.3
	50–74%		23	29.5		17	22.7
	< 50%		10	12.8		13	17.3
	Not available		4	5.1		5	6.7
**Follow-up clinical outcome**	> 95%		14	18.4		9	12.0
	75–95%		18	23.7		21	28.0
	50–74%		25	32.9		22	29.3
	< 50%		14	18.4		17	22.7
	Not available		5	6.6		6	8.0
**Behavioral change counseling**	> 95%		9	11.7		7	9.3
	75–95%		16	20.8		19	25.3
	50–74%		25	32.5		20	26.7
	< 50%		21	27.3		22	29.3
	Not available		6	7.8		7	9.3
**Continuity of medication**	> 95%		19	24.7		16	21.3
	75–95%		33	42.9		31	41.3
	50–74%		13	16.9		13	17.3
	< 50%		8	10.4		10	13.3
	Not available		4	5.2		5	6.7
**Home visit**	> 95%		5	6.5		3	4.1
	75–95%		11	14.3		11	15.1
	50–74%		20	26.0		24	32.9
	< 50%		30	39.0		27	37
	Not available		11	14.3		8	11.0
**NCD prevention in community**	> 95%		5	6.8		4	5.5
	75–95%		14	18.9		10	13.7
	50–74%		19	25.7		19	26.0
	< 50%		22	29.7		24	32.9
	Not available		14	18.9		16	21.9

### Phase II

In phase II, fourteen key informants were purposively selected from three levels of healthcare facilities, primary care, secondary care, and tertiary care from the National Health Security Office (NHSO). All key informants are the directors of the selected healthcare facilities, and the NCD-related division of NHSO, as well as physicians and nurses who were in charge of the NCD clinic (Table 4).

**Table 4:** Job Titles of Key Informants (n = 13)

Job title	Number
Directors/Deputy directors	4
Head of Out-patient Division	2
Physicians who are in charge of the DM clinics	4
Nurses who are in charge of the DM clinics	3

The contents of the in-depth interviews were thematically analyzed in order to understand the impact of COVID-19 on healthcare facilities, and the service resiliency of the healthcare facilities in Bangkok.

### Impacts of COVID-19 on NCD services

All key informants consistently mentioned the effects of the vast number of COVID-19 patients, and public health measures including social distancing, travel restrictions, and lock-downs, on NCD services. COVID-19 greater impacted healthcare facilities which are facing a shortage of healthcare personnel.

*“Our hospital is extremely overwhelmed by COVID-19 because we are the only hospital in this area…number of COVID-19 infected cases is extremely high every day… some staff are infected with COVID-19 and cause ineffective services… we had to deploy NCD staff to support COVID-19 efforts…stop providing some services such as NCD screening in the community, and home visits” (Director of secondary care #1, Head of Out-patient Division #1, Directors of health centers #1 and #2)*.

*“Due to social distancing, we cannot provide services as usual…we had to brainstorm among staff how can we continue our services to serve the same number of patients who need medical care each day…” (Director of Health Center #1)*.

*“Many patients were lost from a follow-up appointment…some of them scared of the COVID-19 transmission and some complied with the national measures” (NCD Physician of Tertiary care #1, Director of secondary care #1)*.

*“We have only one nurse who is taking care of all NCD clinics…the authority had to pool human resource to fight against COVID-19…we just keep the service moving…” (NCD Physician of Secondary care #2)*.

Also, the national policy to fight against COVID-19 such as vaccination, case investigation, and active case findings affected the services of healthcare facilities in particular those BMA health centers. The BMA health centers provide both public health and medical services, the health centers’ staff also involve in COVID-19 active case finding, case investigation, and proactive vaccine services in the community. Therefore, most of the health centers struggled with service providing COVID-19 transmission.

*“As you may know, we have two arms, one arm does medical service and another are does prevention and control diseases …so our staff are frontline staff to fight against COVID-19 in Bangkok…” (Director of Health Center #1, #2)*.

### The resilience capacity at the healthcare facilities

The resilience capacity of the healthcare facilities was analyzed and presented in three themes, absorptive capacity, adaptive capacity, and transformative capacity.

#### Absorptive capacity

We found different responses to the impacts of COVID-19 between secondary and tertiary care facilities and the BMA health centers. With limited human resources and extreme workloads to mitigate the COVID-19 pandemic in Bangkok, the health care facilities extended their service time and applied the common social media application such as LINE application to accommodate patient needs such as pre-appointment service, and health education.

*“Our health center has at least 200 cases per day but we have only 50% health personnel during the COVID-19 pandemic because others fully work for COVID-19 relief…we have to extend our service time to accommodate the needs of our patients…: (Director, BMA health center#1)*

*“We cannot focus on the NCD clinics, our area has been extremely overwhelmed by COVID-19…we have to put our effort on that…” (Nurse, BMA health center#2)*.

#### Adaptive capacity

Drive-through and Fill-in corners are two alternative pharmacy services provided for well-controlled DM patients in order to provide accessibility to medication and COVID-19 risk reduction. Well-controlled DM patients can submit the request of the Drive-through or Fill-in corner to the healthcare facilities and come to pick up the medication at the appointment time.

*“Our patients can come to the Fill-in corner…they just make an appointment with the physician to monitor and investigate the patient’s current health status via our application and then come any time to pick up the medication…” (Physician, Tertiary care hospital#1)*.

However, one tertiary care facility only applied the LINE application to provide health education or counseling. This healthcare facility provides comprehensive DM care and services on specific days, DM type I clinic, Poor-Controlled DM clinic, Well-Controlled DM clinic, and Health Education and Counseling clinic for high DM risk groups and DM family members. The comprehensive DM care and services for each harmonized specific group allow the healthcare facility to internally formulate the resource plan while optimally customizing the service times for patients.

*“Although, our DM staff have been allocated to fight against the COVID-19 pandemic, our clinic still offers the service as usual…we classified patients into 4 groups that makes us easily manage flow of patients each day…therefore we can efficiently manage our service even though the lessening numbers of staff…” (Nurse, Tertiary care hospital#2)*.

#### Transformative capacity

The healthcare facilities under BMA collaborated with the NHSO to provide three new services; (1) mobile laboratory (2) medication delivery and refilled corner at drug store, and (3) Mobile health (mhealth). These three services will be financially supported by the NHSO exclusively for patients under the universal coverage scheme (UCS).

The mobile laboratory is a service that the BMA collaborated with the private sector which works to collect blood specimens at patients’ houses and send them back to the hospital laboratory. Surprisingly, we found only two among six healthcare facilities offered the mobile laboratory to their patients due to the limited boundary of the services. The healthcare facilities located at the skirt of Bangkok utilized this service less because their patients majorly come from the neighboring areas of Bangkok.

*“Mobile laboratory is not well accepted among our patients because they live in this neighborhood and they can come for specimen process conveniently…” (Director, health center#1 and #2)*.

*“Our patient can use not only the mobile laboratory but also Self-Monitoring of Blood Glucose using glycemic reader devices…and uploaded the results on the LINE OA…” (Nurse, Tertiary care hospital# 1)*.

Together with the mobile laboratory, the mHealth application is also widely implemented in Bangkok and Thailand. The BMA and the Department of Medical service launch the mHealth application for their healthcare facilities, MorBMA (“Mor” is Thai and means physician), and DMS telemedicine application, respectively. All healthcare facilities used the application to continually provide service to their well-controlled blood sugar levels patients. DM patients can upload the results of blood glucose tests via LINE or mHealth application. On the follow-up date, patients connect to the application and virtually see the physician. Because of the higher number of elderly DM patients, the elderly may not be familiar with the mHealth therefore the healthcare facilities identify a caretaker from family, community, or health center to assist the elderly patient in successfully utilizing mHealth. Together with mHealth, the healthcare services offered medication delivery and medication refills at the drug store. The healthcare facilities collaborated with the delivery industry such as Thailand Post, and other private delivery industries. The service fee is either paid by the NHSO or the patient’s out-of-pocket expense. The DM patients can also pick up the medication at the drug store in the healthcare facilities neighborhood. The drug stores in the neighborhood must be pre-registered with the NHSO and the healthcare facilities before operating this service.

*“our hospital plans to implement the mHealth before the COVID-19…I can say that the COVID-19 escalates the speed of our implementation…” (Deputy director, tertiary care hospital#1)*.

*“We have been facing with the staff shortage, mHealth is one approach that I am trying to implement…COVID-19 can help me to escalate acceptability among staff…change comfortable routine service to something new may be difficult but it is worth to do…” (Director, secondary care hospital #1, and Director, health center#1)*.

*“I can observe the increased utilization trend of DMS application and medication delivery services among DM patients…however, the stability of the mHealth system is remained a challenge …” (Physician, tertiary care hospital#1)*.

*“Yes, some patients don’t know how to use LINE or mHealth application, nurses as us must explore the care takers from family, community, and health centers…healthcare network and collaboration with community is importance for us…”(Nurse, tertiary care hospital#2 and Nurse, secondary care hospital#2)*.

We also found that one tertiary care hospital collaborated with academic and research institutes to innovate a medication application that can improve the safe use of medication for chronic illness patient like DM, namely Pharmasafe, which can support the family member and the healthcare facility to monitor compliance and reduce drug interactions in patients.

### Crucial success factors

During the COVID-19 epidemic in Bangkok, the healthcare facilities continued the DM services in regards to three periods of the service adaptation. The crucial factors for successful adaptation can be classified into two domains, inner context and outer context.

#### Inner context

Leadership among healthcare personnel at each level is the most crucial success factors. We observed strong policyand decision making among the healthcare facilities’ director. In three out of six healthcare facilities, the directors arranged implementation plans and used strategic communication to penetrate comfortable routine services and utilize innovative services such as mHealth and Drive-through service. Also, nurses at the DM clinic play an important role in accommodating new services for patient’s convenience. Leadership among nurses at the DM clinic was also expressed via the knowledge transferring plan among senior and junior staff. This organizations culture allows nurses in the DM clinic to do other work that appropriately and strictly follows the core values of the organization to achieve the implementation even during the limited resource period.

The mHealth can quickly be implemented because of the how well established each healthcare facility is. For instance, a DM patient from the tertiary care hospital made a text inquiry about food and medication via the LINE application, the nurse can check the hospital ID and identify the clinical status, responsible physician, and provide sufficient information for nutritionist and pharmacist to respond to the patient’s inquiries. However, the different health information systems and lack of collaboration across healthcare facilities limit service accessibility during the crisis.

The adequacy of the healthcare personnel during the normal operations is important. We found that one hospital has been dealing with an inadequate number of personnel at the DM clinic, therefore, during the COVID-19 crisis, this issue is exacerbated and has led to job fatigue, and exhausted among the healthcare personnel.

*“We have only one nurse at DM clinic…during the COVID-19 she has to do other works at other clinics…how she can care patient properly…we can do at our limit…” (Physician, secondary care hospital #2)*.

#### Outer context

The BMA and the NHSO policy fully drives the healthcare facility to operate healthcare service during the COVID-19 situation. NHSO adapted reimbursement policy for chronic illness patients, and financially supported three innovative services, i.e. mHealth, mobile laboratory, medication delivery and refills at drug stores. This is likely a breakthrough in the limit of service and regulation that will increase accessibility to continuum care among patients with chronic illnesses, such as DM.

During the public health crisis, transportation is limited and is a huge obstacle for patients. Thus, a Partnership with private clinic networks close to the patient’s community to provide service for those well-controlled blood glucose level, community-based organization to operate community-based practice such as home visit, and home-based education, plays an important role in access to care. We found that the hospitals that have well defined partners can provide continuum care with their patients during the crisis to same capacity as before the Covid-19 situation.

*“We cannot operate home visit, so we contact our partners/network that locate close to the patient to operate home visit for the necessitate cases…yes, of course we must communicate both sides about the operation and our partner then report to us…we then record into the health information system to complete all activities with the patients…the physician or DM team can use this information to customize the treatment and care…” (Nurses, tertiary care hospital #2, and secondary care hospital #2, Director, secondary care hospital#2)*.

## Discussion

The impact of COVID-19 on NCD service disruptions in the second wave (2021)wasmore severe than the first wave (2020). The main reasons of NCD service disruptions are insufficient staff and closure of hospital services. Surprisingly, both the hospital budget and medical supply among healthcare facilities in Bangkok are less affected by the COVID-19 pandemic. Our qualitative findings revealed resilience capability i.e. absorptive, adaptive, transformative capability, among the healthcare facilities that provide continuum care by increasing availability and accessibility to healthcare services among patients with chronic illness, such as DM.

Not surprisingly, primary healthcare facilities, which is closer to community than secondary and tertiary healthcare facilities, commonly expressed absorptive capability by using the existing resources such as working hours management while higher level of healthcare expressed adaptive and transformative capabilities.

In this study, we found the strategies to adapt the healthcare services for NCD patients during the public health crisis are firstly, leveraging affordable and common digital technology to increase virtual follow-up visit, tele-consultation, tele-monitoring and tele-pharmacy. This strategy also recommended in previous studies in the emergency division [22–28]. Maintaining follow-up visits among DM patient using digital technologies in this study is consistent with the study in Brazil where a majority of the patients were followed-up and had their glycemic levels monitored remotely via telemedicine [23]. The Significance of telemedicine on better glycemic control among DM type was reported in the Saudi Arabian study [27, 28]. Providing mHealth or telemedicine may greatly improve the healthcare services for DM patients, if the healthcare facilities can link the health information system or individual health data to the application. To do so, the healthcare facilities in Bangkok can provide seamless optimal care to DM patients as a result of having access to the clinical data with patients consents.

The second strategy found in this study is to transform the existing route of services to penetrate the hard-to-reach area such as mobile laboratories, Drive-through service, medication delivery and refilling at the drug store, and collaboration with the private sectors and civil society to ensure all patients can conveniently access service in the long run. Consistent prescribed medication and glycemic monitoring are two crucial services for DM management [3, 29].

The third strategy links with absorptive capabilities, the healthcare facilities utilizes the existing resources to enhance less specialized healthcare personnel to deliver online health education, behavioral change counseling, and online training on self-care procedures such as using a device for self-monitoring of blood glucose. This strategy can mitigate the mental health impact of COVID-19 which a study in India reported mental health, and decreased in the self-monitoring glycemic among DM patients [29]. Providing consistent communication for DM patients by healthcare providers can lessen loneliness and encourage self-monitoring among DM patients. Finally, the political-will can ensure financial support the transformation of healthcare services.

This study has some limitations, firstly we conducted this study during the COVID-19 pandemic, all interviews were conducted via the online platform, and the service observation was done in one healthcare facility only. However, we requested the healthcare facilities to send photos of up-to-date circumstances of the DM services to the research team. We did not measure the clinical outcomes of DM patients in six study settings. This study described only the provider perspective. Due to the large portion of non-respondents among university hospital, private hospitals and hospitals under the jurisdiction of other ministry of defense, and finance, the findings can generalize to the health facilities under jurisdiction of the Bangkok Metropolitan which is the key stakeholders of healthcare services in Bangkok.

## Conclusion

This study emphasized the DM service models to continually provide services, and crucial resilience capabilities of healthcare facilities during the public health crisis. Using affordable and common digital technologies to ensure DM patients can access continuum care and providing alternative services for medical laboratories, and medication delivery and refill at drug stores can increase consistent monitoring of glycemic levels and use of prescribed medication.

## Data Availability

The datasets generated and/or analyzed during the current study are not publicly available due to ethical thought but are available from the corresponding author on reasonable request.

## List of Abbreviations

BMA: Bangkok Metropolitan Administration
DM: Diabetes mellitus
NCD: Noncommunicable diseases
PCC: Primary Care Commissioning Cluster
WHO: World Health organization
